# Chromatin-associated ncRNA activities

**DOI:** 10.1007/s10577-013-9390-8

**Published:** 2013-11-19

**Authors:** Claudia Keller, Marc Bühler

**Affiliations:** 1 Friedrich Miescher Institute for Biomedical Research, Maulbeerstrasse 66, 4058 Basel, Switzerland; 2 University of Basel, Petersplatz 10, 4003 Basel, Switzerland

**Keywords:** non-coding RNA, chromatin, genome regulation

## Abstract

RNA transcripts that do not code for proteins have been long known to lie at the heart of many biological processes, such as splicing and translation. Yet their full potential has only been appreciated recently and non-coding RNAs (ncRNAs) are now attracting increasing attention. Pioneering work in yeast and plant systems has revealed that non-coding RNAs can have a major influence on the deposition of histone and DNA modifications. This can introduce heritable variation into gene expression and, thus, be the basis of epigenetic phenomena. Mechanistically, such processes have been studied extensively in the fission yeast *Schizosaccharomyces pombe*, providing an important conceptual framework for possible modes of action of ncRNAs also in other organisms. In this review, we highlight mechanistic insights into chromatin-associated ncRNA activities gained from work with fission yeast, and we draw parallels to studies in other eukaryotes that indicate evolutionary conservation.

## Introduction

Recent advances in technologies enabling the profiling of transcriptomes at unprecedented depth have revealed the production of RNA in eukaryotic cells from a proportion of their genomes much larger than anticipated (Guttman et al. 2009, 2011; Willingham et al. 2006; Cheng et al. 2005; Birney et al. 2007; Kapranov et al. 2007a; Wilhelm et al. 2008). Estimates from the ENCODE consortium suggest that more than three quarters of the human genome is transcriptionally active (Djebali et al. 2012; Dunham et al. 2012), with the number of transcripts detected by far exceeding the number of known mRNAs coding for proteins. Whereas such non-protein coding RNA (ncRNA) catalogs are increasing at a great pace, the extent of ncRNA involvement in regulatory circuits and the mechanisms by which they might function remain largely unexplored. Furthermore, questions remain about what portion of the non-coding transcriptome is functionally relevant or simply reflects transcriptional “noise” (Struhl 2007). In particular, the observations that ncRNAs from genomic regions thought to be transcriptionally silent or from intergenic or promoter regions are often subjected to rapid degradation (Keller et al. 2012; Lubas et al. 2011; Houseley et al. 2007; Wyers et al. 2005; Vasiljeva et al. 2008; LaCava et al. 2005; Preker et al. 2008; Buhler et al. 2007; Woolcock et al. 2010), and that most ncRNAs are repressed below one copy per cell (Marguerat et al. 2012) have led to the hypothesis that these transcript are a consequence of an imperfect transcription machinery that produces spurious RNAs. Indeed, estimates of the fraction of the vast amount of ncRNAs that might be physiologically relevant differ considerably (Kowalczyk et al. 2012). Thus, biologists should be urged to dissect the various putative modes of action of ncRNAs, which is much more laborious and challenging than cataloguing but is essential for a solid comprehension of the non-protein coding genome.

Non-coding RNAs are typically classified into small and long ncRNAs based on an artificial length threshold of about 200 nucleotides (nt) (Kapranov et al. 2007b); this value simply reflects the nucleic acid absorption cutoff of the matrices most commonly used to isolate RNA molecules. Small ncRNAs include the well-studied transfer (t)RNAs, small nucleolar (sno)RNAs, small nuclear (sn)RNAs and micro (mi)RNAs, which range in size from 20 nt for miRNAs up to approximately 150 nt for snRNAs (Ma et al. 2013). Long ncRNAs (>200 nt) have received less attention, but a few examples have revealed that they can exert crucial functions in both the cytoplasmic and nuclear compartments of a cell. Intriguingly, there is growing evidence that ncRNAs are actively involved in genome regulation in various eukaryotes. Work with yeast and plant systems has revealed ncRNAs that have a major influence on the deposition of histone or on DNA modification, which can introduce heritable variation of gene expression without altering the DNA sequence and, thus, lead to epigenetic phenomena (Hall et al. 2002; Heo and Sung 2010; Herr et al. 2005; Onodera et al. 2005; Verdel et al. 2004). In particular, the fission yeast *Schizosaccharomyces pombe* has served as a tremendously powerful model organism to investigate the links between ncRNAs and chromatin at a mechanistic level, establishing guiding paradigms for studying ncRNA-mediated genome regulation in other eukaryotes. In this review, we focus on mechanistic insights into chromatin-associated ncRNA activities that have been gained with *S*. *pombe* and draw parallels to studies in other organisms that indicate evolutionary conservation.

## ncRNAs acting as guide molecules

**Fig. 1:**
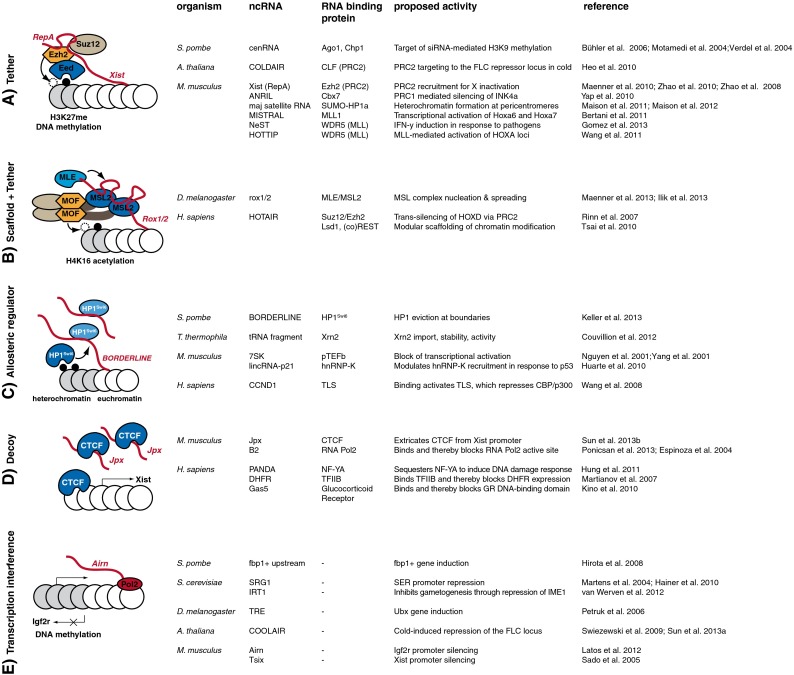
Various chromatin-associated activities of long ncRNAs. (*A*) ncRNA tethers link activities in *cis* via a nascent transcript. For example, the ncRNA Xist is transcribed from the X-chromosome and interacts via a conserved stem-loop sequence termed RepA with the PRC2 complex members Ezh2 and Suz12. This triggers H3K27 methylation, which is recognized by the H3K27 reader Eed. This leads to inactivation of the X chromosome. (*B*) In addition to acting as tethers, some long ncRNAs may also act as scaffolds. The ncRNA (e.g. rox1/2) not only serves as a nucleation site but is also an integral part of the chromatin-modifying activity (e.g. the MSL complex). In the case of the *Drosophila* MSL complex, this leads to H4K16 acetylation via the histone acetyltransferase MOF and transcriptional upregulation on the male X chromosome. (*C*) ncRNAs acting as allosteric regulators affect chromatin effectors by direct binding and tuning of their molecular properties. For example, RNA binding to the hinge region of HP1^Swi6^ induces conformational changes in the HP1^Swi6^ CD, which is incompatible with stable H3K9me3 association. This leads ultimately to HP1^Swi6^ eviction from heterochromatin. (*D*) Decoys mimicking a natural ligand of the molecule to be regulated compete for the binding site. For example, Jpx RNA contains a motif that effectively competes with CTCF DNA binding. This extricates CTCF from the Xist promoter and thereby activates Xist transcription, inducing X chromosome inactivation in female mice. (*E*) In the transcriptional interference model, the action of the transcribing RNA polymerase rather than the RNA product is functionally relevant. One example is transcriptional overlap of the Airn ncRNA, which is required for Igf2r promoter silencing. The table lists representative examples of each mode of action

Arguably, the best established attribute of ncRNAs is that they can guide associated partner proteins to other nucleic acid target molecules by complementary base-pairing. In particular, small ncRNAs guide enzymatic activities to targets, endowing specificity on pathways that, for example, cleave and ligate RNA, modify RNA or DNA, regulate telomere length, or modify chromatin. Extensively studied examples of RNA guides are snRNAs, snoRNAs and tRNAs, which are crucial to the specificity of pre-mRNA splicing, the 2′-O-ribosylation and pseudo-uridylation of ribosomal RNA, or the decoding of the open reading frame of an mRNA during protein synthesis, respectively (Hopper and Phizicky 2003; Matera et al. 2007). An important feature of snRNAs and snoRNAs is that they contain several sequence motifs and RNA secondary structures that act as binding sites for specific partner proteins in *cis*, in addition to a *trans*-acting guide element (Matera et al. 2007). Thus, snoRNAs and snRNAs act at the same time as scaffolds assembling specific ribonucleoprotein (RNP) complexes and as guide molecules. We envision that this principle also applies to many of the recently discovered long ncRNAs (Fig. 1).

In contrast, small ncRNAs that function in RNA interference (RNAi)-related pathways act solely as guide molecules (Hannon 2002). With a size range of 20–31 nt, depending on the type of small RNA and organism, they are simply too short to also encode scaffolding motifs. However, we note that such elements do exist in the precursors of the small RNAs and are important for their maturation (Yates et al. 2013).

**Fig. 2:**
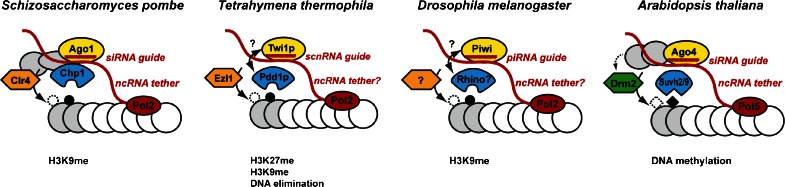
Small and long ncRNAs cooperate in the localization of effector complexes to chromatin. In *S*. *pombe*, siRNAs guide the RITS complex consisting of the Argonaute protein Ago1 (*yellow*), the chromodomain (CD) protein Chp1 (*blue*) and Tas3 (*grey*) to nascent transcripts originating from centromeric repeat sequences; this is followed by recruitment of the H3K9 methyltransferase Clr4. The CD of Chp1 recognizes H3K9-methylated histone tails and thereby stabilizes the complex on chromatin. The Chp1 CD also possesses intrinsic affinity for nucleic acids, which might further stabilize the complex on chromatin. In *Tetrahymena thermophila*, the Argonaute protein Twi1p is similarly involved in the recruitment of the H3K27me reader protein Pdd1p and the H3K27-methyltransferase Ezl1 via a 28-nt scnRNA guide. Whether this also involves nascent transcript interactions through a RITS-like complex remains to be shown. Ultimately, this process leads to DNA elimination. In *Drosophila*, Piwi interacts with a piRNA guide, which targets transposable elements (TEs) for transcriptional silencing via H3K9 methylation. Although direct evidence is still awaited, nascent TE transcripts are also thought to function as assembly platforms. The CD-protein Rhino has been implicated in piRNA generation. In *Arabidopsis thaliana*, Ago4-associated small RNAs guide the DNA methylation machinery to target sites. Ago4 can interact with nascent transcripts generated by RNA Polymerase 5 (Pol5) as well as with Pol5 itself

RNAi, first recognized as a double-stranded RNA (dsRNA)-mediated process in *Caenorhabditis elegans* in 1998 (Fire et al. 1998), exists in various forms in a wide variety of eukaryotic organisms (Ghildiyal and Zamore 2009). A central feature of RNAi are the *trans*-acting Argonaute proteins that are guided to complementary targets by their bound small RNAs (Fig. 2) (Joshua-Tor and Hannon 2010; Meister 2013). Notably, Argonaute can be programmed with any small RNA guide and will target, in principle, any complementary sequence, may this be endogenous or exogenous (Brummelkamp et al. 2002; Elbashir et al. 2001). Therefore, RNAi has been widely exploited as an experimental technique and is considered to have great potential for therapeutics (Davidson and McCray 2011). The enormous impact on biotechnology and the still emerging physiological roles of small ncRNAs showcase the extraordinary power of ncRNAs in guiding protein complexes to their destination.

## Small ncRNA-mediated guidance of effector complexes to chromatin

Whereas miRNAs are well-known guide molecules that act at a post-transcriptional level in the cytoplasm (Guo et al. 2010), small ncRNA guides are known to function in the nucleus in some organisms, specifying the targeting of protein effector complexes to their respective chromosomal loci. Classical examples are the targeting of histone or DNA methylation activities to chromatin, but the directing of other activities to chromatin may be equally possible.

RNA-mediated chromatin modification was first observed in plants (Wassenegger et al. 1994). Although the mechanism was not understood at the time, it is now known that small ncRNAs play a key role in RNA-directed DNA methylation (RdDM). Repetitive genomic sequences, including transposons and centromeric repeats, produce 24-nt siRNAs that target DNA methylation, silencing these regions and other loci homologous to the siRNAs (Matzke et al. 2009; Zhang and Zhu 2011). More recently, 21-nt small ncRNAs (diRNAs) produced from DNA double-stranded breaks (DSBs) have been proposed to guide Ago2 to damaged chromatin and mediate repair in *Arabidopsis* (Wei et al. 2012). Similarly, small ncRNAs (ddRNAs) have been linked to the DNA-damage response in humans, mouse and zebrafish (Francia et al. 2012; Wei et al. 2012). This is reminiscent of earlier studies in the filamentous fungus *Neurospora crassa*, where 20- to 21-nt ncRNAs (qiRNA) generated upon DNA damage were proposed to affect protein synthesis after DNA damage by inhibiting rRNA biogenesis (Lee et al. 2009). It is tempting to speculate that these small ncRNAs are actively involved in DNA repair also by guiding protein factors to the damaged site, an intriguing model that deserves further investigation in these organisms.

Small ncRNA guides in ciliated protozoa have also been directly linked to chromatin (Mochizuki 2011; Bracht et al. 2013). In *Tetrahymena thermophila*, an extreme example of RNAi-directed heterochromatin formation leads eventually to DNA elimination of transposon-related sequences from the newly developing somatic (macronuclear) genome (Kataoka and Mochizuki 2011). During sexual conjugation, the whole germline (micronuclear) genome is bidirectionally transcribed and processed in the nucleus to 28- to 29-nt “scan” RNAs (scnRNAs) (Malone et al. 2005; Mochizuki and Gorovsky 2005). These scnRNAs associate with an Argonaute protein and translocate to the parental macronucleus, where it is thought that base-pairing interactions between the scnRNAs and nascent non-coding transcripts from the parental macronuclear genome mediate scnRNA degradation. Therefore, this ‘scnRNA selection’ leaves behind only scnRNAs for which there are no homologous sequences in the parental macronuclear genome. The remaining scnRNAs, still bound to Argonaute, relocate to the newly developing macronucleus, where they target homologous sequences and mediate methylation of histone H3 lysine 9 and 27 (H3K9 and H3K27, respectively). Subsequent binding of chromodomain proteins marks these regions, which leads to excision by a PiggyBac transposase-like protein (Cheng et al. 2010). Therefore, this elegant ncRNA-mediated mechanism eliminates any sequences not present in the parental macronucleus (Fig. 2). Intriguingly, small ncRNAs in another ciliate, *Oxytricha trifallax*, also act in genome rearrangement but instead of eliminating they specify which sequences are retained (Bracht et al. 2013).

Furthermore, the more recently discovered piwi-interacting (pi)RNAs that control transposable elements in the germline of animals have been linked to chromatin regulatory processes. Mutations in piRNA pathway components led to defective DNA methylation in male mouse germ cells (Pillai and Chuma 2012; Aravin et al. 2006, 2008; Carmell et al. 2007; O’Donnell and Boeke 2007) and reduced H3K9 methylation at transposon loci in *Drosophila* ovarian somatic cells (Sienski et al. 2012; Rozhkov et al. 2013; Le Thomas et al. 2013). Finally, in *C. elegans*, artificially introduced or endogenous small ncRNAs can silence complementary, nuclear-retained RNAs or nuclear-localized polycistronic RNAs. This is accompanied by increased H3K9 methylation of the underlying chromosomal locus. These observations strongly argue that small ncRNAs can guide the H3K9 methylation machinery to chromatin also in worms (Burkhart et al. 2011; Burton et al. 2011; Guang et al. 2010; Lee et al. 2012; Bagijn et al. 2012)

Emerging mechanistic models for many of the above-mentioned examples are reminiscent of the basic concepts that arose from studies of RNAi-mediated heterochromatin formation in the fission yeast *S*. *pombe*, which is one of the most intensely studied examples of small ncRNA-guided chromatin modification. A decade ago, it was shown that all three major components of the RNAi machinery in *S*. *pombe*, namely Argonaute (Ago1), Dicer (Dcr1) and the RNA-dependent RNA polymerase (Rdp1), are essential for the formation of centromeric heterochromatin (Volpe et al. 2002; Provost et al. 2002) and small ncRNAs were identified that map to this supposedly transcriptionally inactive chromatin structure (Reinhart and Bartel 2002). Meanwhile, a number of protein complexes involved in this process have been identified (Buhler 2009; Lejeune and Allshire 2011; Reyes-Turcu and Grewal 2012; Moazed 2009; Castel and Martienssen 2013) and their intense study has provided solid evidence for a small ncRNA-guided model for chromatin modification.

In brief, the RNA-induced transcriptional silencing complex (RITS; consisting of Ago1, Chp1 and Tas3) (Verdel et al. 2004) accepts Dcr1-dependent single-stranded siRNAs that guide RITS to chromatin, where it recruits CLRC, a complex containing the sole *S*. *pombe* H3K9 methyltransferase Clr4 (Bayne et al. 2010). Similar to the situation in *Tetrahymena*, this RNAi-mediated histone methylation provides a binding site for HP1 homologues, such as Swi6 and Chp2, and can also stabilize binding of RITS via the chromodomain (CD)-containing Chp1 component. Interestingly, the Chp1 CD also possesses intrinsic nucleic acid-binding activity, a property that can further stabilize RITS association with chromatin (Ishida et al. 2012). RITS also helps recruit an Rdp1-containing complex, RDRC, which amplifies the process by generating more double-stranded RNA substrates for Dcr1 (Motamedi et al. 2004; Sugiyama et al. 2007) Thus, the RNAi machinery acts in a positive feedback loop on centromeric repeats, generating high levels of H3K9 methylation and rapid turnover of centromeric RNAs into siRNAs (Buhler and Moazed 2007).

## Long ncRNAs act as binding sites for small ncRNA guides

A general concept for ncRNAs acting in association with chromatin that arose from studies of RNAi-mediated heterochromatin formation in *S*. *pombe* is that small ncRNA guides target the respective chromosomal regions through base-pairing interactions with long, chromatin-associated ncRNAs. Originally proposed by Shiv Grewal and Danesh Moazed (Grewal and Moazed 2003) this conception, also known as the “nascent transcript model”, has been corroborated by several lines of experimental evidence. Firstly, RITS and RDRC associate with centromeric heterochromatin and physically interact with non-coding centromeric RNAs (Motamedi et al. 2004; Noma et al. 2004; Woolcock et al. 2010; Verdel et al. 2004). Secondly, specific mutations in subunits of RNA polymerase II have been identified that do not generally affect transcription but lead to a loss of RNAi-dependent heterochromatin formation (Kato et al. 2005; Djupedal et al. 2005). Finally, direct proof-of-concept for the nascent transcript model was provided by the artificial tethering of RITS to the nascent transcript of a normally euchromatic gene. This was sufficient to trigger the formation of ectopic heterochromatin and activate the characteristic positive feedback loop that secured high levels of H3K9 methylation and efficient silencing of the newly formed heterochromatic locus (Buhler et al. 2006).

Although binding of the siRNA guide to single-stranded DNA cannot be formally ruled out, the experimental evidence for long, chromatin-associated ncRNAs acting as binding sites for small ncRNA guides is substantial. Strong support for this model also arose from studies of RdDM in *Arabidopsis*, which involves the action of two plant-specific RNA polymerases, RNA Pol IV and Pol V (Wierzbicki et al. 2008). Pol IV is thought to be responsible for the transcription of precursor RNAs that are processed by the RNA-dependent RNA polymerase (RdRP) RDR2 and DCL3 (DICER-LIKE 3), producing the 24-nt siRNAs that load onto AGO4 (Herr et al. 2005). Importantly, Pol V is thought to be the RNA polymerase dedicated to the production of transcripts that presumably act as scaffolds in the association of AGO4-siRNA complexes and subsequent chromatin modification (Wierzbicki et al. 2008, 2009) similar to the situation in *S*. *pombe*. Finally, scnRNAs in *Tetrahymena* and piRNAs in *Drosophila* and *C*. *elegans* are also thought to guide their associated Argonaute/Piwi proteins to the respective chromosomal loci via base-pairing with nascent transcripts (Luteijn and Ketting 2013).

## Long ncRNAs acting as tethers: a recurrent theme

The nascent transcript model for small ncRNA-mediated chromatin regulation described above is reminiscent of proposals put forward to explain the role of long ncRNAs in X chromosomal dosage compensation in *Drosophila* and mouse. In flies, this process involves upregulated transcription on the male X chromosome via recruitment of the MSL complex, which deposits histone H4K16 acetylation (Conrad and Akhtar 2012). Following the discovery that the roX1 ncRNA specifically coats the X-chromosome, it was proposed more than a decade ago that ncRNAs change chromatin conformation by associating with MSL complexes, histone acetyltransferases, or chromatin components (Meller et al. 1997). Later, it was demonstrated that roX ncRNAs are an integral part of the MSL complex and thereby exert a scaffolding activity (Meller et al. 2000; Smith et al. 2000; Akhtar et al. 2000).

In vivo, the two functionally redundant ncRNAs roX1 and roX2 are encoded on the X chromosome itself. Only the roX1/2 double mutant results in male-specific lethality. This phenotype can be rescued by autosomal roX transgenes, which demonstrates that the roX1/2 lncRNAs can also function in *trans* (Meller and Rattner 2002). Autosomally expressed roX transgenes can trigger assembly and spreading of the MSL complex on the ectopic locus, but only if the X-linked roX sequences are mutated at the same time (Park et al. 2002). The roX RNAs, therefore, seem to compete for limiting MSL complexes and preferentially target the X chromosome, a property that may be provided by specific DNA sequence motifs (Alekseyenko et al. 2008). Recent iCLIP data indeed has confirmed that the MSL members MLE and MSL2 are involved in co-transcriptional incorporation of the roX ncRNAs into the MSL complex, which results in X-specific nucleation and spreading (Maenner et al. 2013; Park et al. 2002; Ilik et al. 2013). Taken together, the available data suggest that roX ncRNAs exert both scaffolding and *cis*-specific tethering activities.

In mammals, the mechanism of dosage compensation is fundamentally different in that it manifests in females, where one of the two X chromosomes is epigenetically silenced via H3K27me and DNA methylation. However, similar to the situation in *Drosophila*, a number of ncRNAs are involved in this process, with Xist being the most prominent example. When the Xist gene was identified about 20 years ago, it was noted that Xist RNA expression paradoxically occurs exclusively from the inactive X chromosome in females (Brown et al. 1991; Borsani et al. 1991; Brockdorff et al. 1991). While it was conceivable that Xist encodes for a protein (Borsani et al. 1991), Brown et al. hypothesized that “the XIST product is a *cis*-acting RNA molecule, perhaps involved structurally in the formation of the heterochromatic Barr body” (Brown et al. 1991). This hypothesis is supported by the fact that Xist contains no significant open reading frame, is not associated with the translational machinery, localizes predominantly to the nucleus (Brockdorff et al. 1992; Brown et al. 1992), and acts in a *cis*-specific manner (Penny et al. 1996). Brockdorff and colleagues proposed that Xist RNA molecules might (a) locally interact with chromatin, (b) form a complex with other nuclear factors, which might in turn interact with chromatin, or (c) change the subnuclear localization of the X chromosome, which eventually results in heterochromatinization. Alternatively, they suggested (d) that active transcription through the Xist locus, and not necessarily the Xist product, causes a change in chromatin structure (Brown et al. 1992; Brockdorff et al. 1992). Accordingly, autosomal expression of Xist is sufficient to mediate chromosome-wide changes and silence the transgenic chromosome (Lee et al. 1996; Lee and Jaenisch 1997; Wutz and Jaenisch 2000). Mapping experiments have demonstrated that the domains responsible for silencing lie within the 5′ repeat A (RepA). RepA folds into stem loops, which were proposed to act as binding sites for factors involved in heterochromatin formation (Wutz et al. 2002). These initial hypotheses gained further support recently when RepA was shown to bind to the PRC2 complex member Ezh2 (Zhao et al. 2008; Maenner et al. 2010; Kaneko et al. 2010). RepA might thereby recruit PRC2, which then triggers H3K27me and silencing of the X chromosome in *cis*.

Interestingly, apart from Xist, ncRNAs could play a more general role in PRC2 recruitment to chromatin. For example HOTAIR, a 2.2-kb ncRNA expressed at the boundary of two chromatin domains within the HOXC cluster, was shown to co-immunopurify with PRC2. Furthermore, knockdown of HOTAIR results in a global loss of H3K27me3 and increased RNA expression at the 40-kb remote HOXD locus. Thus, it is possible that HOTAIR recruits PRC2 to the HOXD cluster. In contrast to Xist, HOTAIR acts in *trans* (Rinn et al. 2007; Tsai et al. 2010). In general, the tethering or recruitment of chromatin-modifying complexes by ncRNAs has been attracting increasing attention. Therefore, it is not surprising that this also includes activating complexes such as the MLL/trithorax complex, which is recruited by the *cis*-acting ncRNA HOTTIP (Wang et al. 2011). We note that in many cases, very little is known about the kinetics and specificity of the proposed RNA–protein interactions, and we believe that generating specific mutations to disrupt the ncRNA–protein interaction will be essential to solve certain controversial issues regarding ncRNA-mediated recruitment of polycomb complexes (Brockdorff 2013; Schorderet and Duboule 2011). Equally valuable are tethering experiments (Buhler et al. 2006; Wang et al. 2011) that will help to define whether or not ncRNAs are sufficient to recruit/tether these complexes to chromatin, and will allow mechanistic dissection of the steps leading to chromatin modification.

## ncRNAs acting as allosteric regulators

Whilst the list of ncRNAs that may function as guides or tethers is ever-expanding, additional paradigms of ncRNA activity are being uncovered. For example, recent work in our lab with *S*. *pombe* disclosed a rather unexpected activity of ncRNAs in regulating association of the heterochromatin protein HP1^Swi6^ with repressive chromatin. Results from a combination of in vivo and in vitro experiments strongly implied that RNA binding to HP1^Swi6^ prevents HP1^Swi6^ from stably associating with chromatin. In particular, we demonstrated that RNA binds directly to HP1^Swi6^, inducing a conformational change in its chromodomain that abolishes the affinity for methylated H3K9 (Buhler and Hiller 2012; Keller et al. 2012). Thus, HP1^Swi6^ binding to RNA is incompatible with stable heterochromatin association and ncRNA molecules can, therefore, evict HP1^Swi6^ from chromatin. This mode of action is functionally relevant for tight repression of heterochromatin and counteracting the spreading of heterochromatin into neighboring euchromatin (Keller et al. 2012, 2013).

Similarly, the ncRNA Jpx was proposed recently to act as an “evictor” of CTCF in mammals (Sun et al. 2013b). X-inactivation in mammals is triggered by transcription of the *cis*-acting long ncRNA Xist (Brockdorff et al. 1992; Brown et al. 1992). The zinc finger protein CTCF represses transcription of the Xist RNA. During the onset of dosage compensation, Jpx RNA is transcribed and binds to CTCF, thereby competing with CTCF binding to the Xist promoter DNA. As a result, Jpx RNA binding to CTCF results in eviction from the Xist promoter and, consequently, activates Xist transcription (Sun et al. 2013b). It remains to be tested whether Jpx RNA functions as an allosteric regulator, inducing a conformational change in CTCF. Alternatively, it is possible that Jpx simply binds the DNA-binding motif of CTCF and thus rather acts as a “decoy”. Similar decoy activities have been described for other mammalian ncRNAs that titrate transcription factors away from their DNA targets (Kino et al. 2010; Martianov et al. 2007).

The RNA-induced conformational change in the chromodomain of HP1^Swi6^ exemplifies the potential of ncRNAs to act as allosteric regulators of their partner proteins, a mode of action of ncRNAs that might turn out to be more prevalent than anticipated. Similar examples of such ncRNA-mediated protein regulation are the B2 or 7SK ncRNAs that are involved in transcriptional control. The murine 178-nt B2 lncRNA is induced in response to heat shock and globally represses transcription. Mechanistically, this is achieved by direct incorporation of the B2 ncRNA into the RNA polymerase II complex, which results in a block of RNA synthesis (Espinoza et al. 2004; Ponicsan et al. 2013). In metazoans, the 7SK ncRNA interacts with and thereby blocks pTEFb, a factor required in the release of proximally paused RNA polymerase II into productive elongation (Nguyen et al. 2001; Yang et al. 2001; Peterlin et al. 2011).

A further noteworthy example of ncRNAs that may fall into the same functional category are 3′ tRNA fragments in *Tetrahymena* that associate with the Argonaute protein Twi12p and are involved in rRNA processing via the exonuclease Xrn2. This is a particularly interesting case in the light of our above discussion of Argonaute-associated small ncRNAs functioning as guide molecules. Rather than acting as a guide, the tRNA fragment was proposed either to change the conformation of Xrn2 or to stabilize, catalytically activate, or import Xrn2 (Couvillion et al. 2012).

## ncRNA-mediated epigenetic changes

A very intriguing aspect of ncRNAs acting on chromatin is, at least to us, the initiation of epigenetic alterations in some instances. For example, the RNAi machinery has been shown to be necessary for the assembly of heterochromatin at the silent mating type locus in *S*. *pombe*. However, once assembled, heterochromatin at the mating type locus is maintained independently of the RNAi pathway (Jia et al. 2004; Hall et al. 2002). Thus, small ncRNAs may constitute one of the signals inducing the formation of a different cellular state that can be maintained and perpetuated over many cell generations, even in the absence of the initial trigger (Ptashne 2007). Notably, the heterochromatic silencing of genes artificially inserted into the silent-mating type locus occurs in a stochastic way and requires a functional RNAi pathway. However, once the repressed state is acquired, it is stably transmitted to future generations through mitosis and meiosis (Noma et al. 2004; Sadaie et al. 2004; Nakayama et al. 2000).

Because ncRNAs would only be involved initially if they were indeed causing alterations in the epigenetic state of a cell, they may not be detected when simply profiling the transcriptome of a given cell. Fortunately, this is not the case for centromeric heterochromatin in *S*. *pombe*, for which RNAi is required to maintain heterochromatin and siRNAs are constantly produced, even though their abundance fluctuates during the cell cycle (Kloc et al. 2008; Chen et al. 2008; Buhler et al. 2008; Volpe et al. 2002). This requirement for RNAi in the maintenance of heterochromatin at centromeres is a property that has been crucial for elucidating the functional role of ncRNAs in heterochromatin formation. If ncRNAs act in other organisms like RNAi at the mating type locus of *S*. *pombe*, there may be many more ncRNA-mediated epigenetic changes to be discovered in the future. Along that line, small ncRNAs have been implicated in transgenerational inheritance of epigenetic traits in flies, plants, mice, and worms (Regulski et al. 2013; Kiani et al. 2013; Rassoulzadegan et al. 2006; Brennecke et al. 2008; Chandler 2010; Ashe et al. 2012; Bagijn et al. 2012; Daxinger and Whitelaw 2012; Shirayama et al. 2012) and it will be exciting to watch this field of research in the coming years.

## Chromatin remodeling and transcriptional interference

As outlined above, there is ample evidence for ncRNAs being directly involved in genome regulation. However, all ncRNAs may not be functionally relevant, even if they appear to be highly abundant. Work on the *S*. *pombe fbp1*+ gene revealed that ncRNA transcription through the promoter region of the *fbp1*+ gene is important for efficient gene activation (Hirota et al. 2008). Notably, insertion of transcription termination sequences into the promoter abolished *fbp1*+ expression and this could not be rescued by expressing the ncRNA ectopically. In contrast, replacing the ncRNA sequence with unrelated sequences lacking terminators did not disturb *fbp1*+ activation. Thus, chromatin remodeling at the promoter region by the transcribing RNA polymerase seems to be important for *fbp1*+ activation. The ncRNA produced can be regarded as a non-functional byproduct in this case.

Similar ncRNA-transcription-dependent but ncRNA-independent regulatory processes have been described in other organisms. In *Saccharomyces cerevisiae*, transcription of the SRG1 ncRNA across the SER3 promoter is required for SER3 repression. It has been suggested that the mechanistic basis for this regulation is the interference of SRG1 transcription with the binding of activators to the SER3 promoter (Martens et al. 2004; Thebault et al. 2011). Similarly, gametogenesis in budding yeast is controlled by the transcription of two ncRNAs. Transcription of the ncRNA IRT1 interferes with expression of the master regulator of IME1 by inhibiting binding of transcription factors to the IME1 promoter (van Werven et al. 2012). In *Drosophila*, intergenic transcription of ncRNAs upstream of the Ultrabithorax (Ubx) gene is linked to Ubx expression. As in the case of SRG1, ncRNA transcription leads to Ubx repression. Finally, extensive truncation and promoter-swap experiments in mice have shown that silencing of the imprinted Ifg2r gene also results from transcriptional overlap between protein-coding and non-coding sequences, without a contribution by the long ncRNA Airn itself (Latos et al. 2012). In conclusion, these examples illustrate that the mere act of transcription and not necessarily the resultant ncRNA can be functionally relevant.

## Perspectives

Much excitement has been triggered by the notion that eukaryotic cells transcribe a much greater proportion of their genomes than anticipated, and that these seemingly non-protein coding RNAs may add yet another layer of complexity to genome regulation. Clearly, the examples highlighted in this review and others demonstrate that some ncRNAs are functionally highly relevant. However, for most of the “non-coding transcriptome” of different organisms, the extent of ncRNA involvement in regulatory circuits and the mechanisms by which they might act remain largely unexplored. Thus, a future challenge will be to systematically probe ncRNAs for physiological relevance.

The power of yeast has been its genetic tractability, which has allowed specific manipulations of an ncRNA and its underlying genomic sequence in order to perform loss-of-function experiments. The ability to do so is of particular importance when the ncRNA under investigation acts in *cis* on chromatin. Given the recent advances in genome-editing technologies (Wang et al. 2013; Hockemeyer et al. 2011; Jinek et al. 2012), similar decisive experiments may sooner or later become more straightforward in mammalian systems. The insertion of premature transcription termination sites, mutation of the ncRNA sequence, or the inclusion of destabilizing elements such as ribozymes into the ncRNA sequence may provide more compelling evidence for the functional relevance of many ncRNAs that have been assigned a physiological role.

Most importantly, the properties and functions of ncRNAs, whether or not their activities are associated with chromatin, are likely to be heavily dependent on their interactions with specific proteins. Therefore, we must aim at developing strategies to determine the protein partners of ncRNAs, similar to a recent proteomic study in which close to 800 proteins were identified based on their ability to bind to polyadenylated transcripts (Baltz et al. 2012). Only when we know with which proteins a particular ncRNA interacts can we start to dissect its mechanism of action precisely.

## Abbreviations

CD: Chromodomain
CLRC: Clr4 methyltransferase complex
CTCF: CCCTC-binding factor
ddRNAs: DICER- and DROSHA-dependent small RNAs
diRNAs: DNA double-stranded break-induced small RNAs
DSB: DNA double-stranded break
dsRNA: double-stranded RNA
HOTAIR: HOX Antisense Intergenic RNA
HP1: Heterochromatin protein 1
iCLIP: Individual-nucleotide resolution UV cross-linking and immunoprecipitation
lncRNA: Long non-coding RNA
miRNAs: Micro RNAs
MLL: Mixed lineage leukemia
MSL: Male-specific lethal
ncRNAs: Non-coding RNAs
Piwi: P-element-induced wimpy testis
piRNAs: Piwi-interacting RNAs
PRC2: Polycomb repressive complex 2
pTEFb: Positive transcription elongation factor b
qiRNAs: QDE-2-interacting small RNAs
RdDM: RNA-directed DNA methylation
RDRC: RNA-directed RNA polymerase complex
RdRP: RNA-dependent RNA polymerase
RITS: RNA-induced transcriptional silencing complex
RNAi: RNA interference
RNP: Ribonucleoprotein
scnRNAs: ‘Scan’ RNAs
snoRNAs: Small nucleolar RNAs
snRNAs: Small nuclear RNAs
tRNAs: Transfer RNAs
Ubx: Ultrabithorax
